# Effect of direct cold atmospheric plasma (diCAP) on microcirculation of intact skin in a controlled mechanical environment

**DOI:** 10.1111/micc.12399

**Published:** 2017-11-06

**Authors:** Thomas Borchardt, Jennifer Ernst, Andreas Helmke, Murat Tanyeli, Arndt F. Schilling, Gunther Felmerer, Wolfgang Viöl

**Affiliations:** ^1^ Department of Sciences and Technology University of Applied Sciences and Arts Goettingen Germany; ^2^ Division of Plastic Surgery Department of Trauma Surgery, Orthopaedics and Plastic Surgery University Medical Center Goettingen Georg‐August‐University Goettingen Germany; ^3^ Application Center for Plasma and Photonic Fraunhofer Institute for Surface Engineering and Thin Films IST Goettingen Germany; ^4^ Department of Trauma Surgery, Orthopaedics and Plastic Surgery University Medical Center Goettingen Georg‐August‐University Goettingen Germany

**Keywords:** blood flow, cold atmospheric plasma, hemoglobin, microcirculation, plasma medicine, skin, tissue oxygen saturation, transcutaneous oxygen pressure, wound healing, wounds

## Abstract

**Objective:**

The microcirculatory response of intact human skin to exposure with diCAP for different durations with a focus on the effect of implied mechanical pressure during plasma treatment was investigated.

**Methods:**

Local relative hemoglobin, blood flow velocity, tissue oxygen saturation, and blood flow were monitored noninvasively for up to 1 hour in 1‐2 mm depth by optical techniques, as well as temperature, pH values, and moisture before and after skin stimulation. The experimental protocol (N = 10) was set up to differentiate between pressure‐ and plasma‐induced effects.

**Results:**

Significant increases in microcirculation were only observed after plasma stimulation but not after pressure stimulus alone. For a period of 1 h after stimulation, local relative hemoglobin was increased by 5.1% after 270 seconds diCAP treatment. Tissue oxygen saturation increased by up to 9.4%, whereas blood flow was doubled (+106%). Skin pH decreased by 0.3 after 180 seconds and 270 seconds diCAP treatment, whereas skin temperature and moisture were not affected.

**Conclusions:**

diCAP treatment of intact skin notably enhances microcirculation for a therapeutically relevant period. This effect is specific to the plasma treatment and not an effect of the applied pressure. Prolonged treatment durations lead to more pronounced effects.

**Abbreviations**
A.U.: arbitrary units
AE: adverse event
BMI: body mass index
CAP: cold atmospheric plasma
DBD: dielectric barrier discharge
diCAP: direct cold atmospheric plasma
O2C: oxygen‐to‐see
rHb: local relative hemoglobin
ROI: region of interest
RONS: reactive oxygen and nitrogen species
SAE: serious adverse event
SDS: tandard deviation
SO2: postcapillary oxygen saturation
*t* test: Student's *t* test
tcPO2: transcutaneous oxygen pressure
TTP: tissue tolerable plasma

## 1 INTRODUCTION

In the context of medicine, the term “plasma” is typically used in conjunction with the uncoagulated cell‐free component of blood. In physics, however, “plasma” refers to the fourth state of matter—ionized gases with unique physical and chemical properties. As multicomponent systems, physical plasmas consist of neutral gas particles, charged particles (ions and electrons) as well as strongly gas‐dependent reactive gas species, and photons. The free electrons in the plasma induce excitation, ionization, and dissociation processes and are mainly responsible for the changes of physico‐chemical properties from inert gases to chemically reactive plasma.

Plasma is usually generated using strong electric fields to induce gas breakdown. As a consequence, electric fields are of major importance for plasma engineering and can be considered as an immanent plasma component. For decades, physical plasmas have been applied in various and versatile processes, for example, to modify surface wettability or deposit functional coatings, for material etching or superficial cleaning and sterilization as well as for radiation generation.^1^ Most of these applications were performed at reduced pressure environments or at process temperatures that would have induced thermal damaging of organic substrates. Technical improvements over the last 15‐20 years have enabled the generation of atmospheric pressure plasmas with gas temperatures as low as room temperature.^2^, ^3^, ^4^, ^5^, ^6^ These CAP, TTP or, in a physically wider sense, nonthermal plasmas have extended the application field from classical inorganic surfaces to organic surfaces and even living tissue. Research and applications in the latter are often referred to as plasma medicine.^7^

Currently available plasma sources can be grouped into direct (diCAP) and indirect sources depending on their characteristic electrode configuration and treatment modalities.^8^, ^9^ For diCAP, the object to be treated is typically part of the electrical circuit and acts as a second or third electrode. As a consequence, the object surface is in direct contact with the active plasma zone and charged species as well as short‐lived may contribute to plasma interaction. For indirect CAP sources, the plasma is typically ignited by a self‐containing electrode configuration inside a cavity or restricted to a surface layer. Thereby, fluxes of charged and short‐lived species to the object surface are smaller and long‐lived species transport may be most relevant to induce biological effects. In order to maintain a necessary constant gas gap between electrode and tissue, some direct sources need to be pressed gently on the tissue surface during application thus inevitably exerting a mechanical pressure on the treated area.

In plasma medicine, several new fields of research have developed, for example, in oncology,^10^, ^11^ in dermatology,^12^, ^13^ and in dentistry.^14^, ^15^ Various effects induced by CAP have been observed. Consistently, a reduction in a wide range of microorganisms was found in vitro and in vivo by groups all over the globe.^16^, ^17^, ^18^, ^19^, ^20^, ^21^, ^22^, ^23^ Further effects include the induction of angiogenesis,^24^ stimulation or inhibition of cell proliferation,^25^ up‐ and downregulation of genes in skin cells,^26^ and virus inactivation.^27^

Recent studies have revealed a significant impact of short‐term diCAP application (up to 90 seconds) on the microcirculation of skin.^28^, ^29^, ^30^ However, in the literature, the role of mechanical pressure induced by the diCAP device was not addressed. As biological tissues are known to react to mechanical forces by, for example, changes in blood flow,^31^ it is so far unclear, if the device‐induced pressure to the tissue should be considered a confounding factor during diCAP application. Furthermore, it is unclear, how the tissue would react to longer treatment times with diCAP. Consequently, in this study, we set out to study the effect of diCAP application for 90 seconds, 180 seconds, and 270 seconds, in a controlled mechanical environment.

## 2 MATERIALS AND METHODS

### 2.1 Ethical approval

The study was approved by the local ethics commission (ethic committee University Medicine Goettingen, 7/8/16) and followed the Declaration of Helsinki. Prior to the experimental participation, the subjects signed the informed written consent.

### 2.2 Participants and ROI

Healthy subjects (N = 10, two female and eight male) aged ≥ 18 years participated in this study. The mean age was 29.0 ± 3.4 (range: 26‐38) years. None of them presented soft tissue injuries or skin inflammation around the area of the tested skin of the dorsal forearm during the whole investigation period. They did not report comorbidities such as vasculitis, diabetes mellitus, chronic kidney or liver disease, or cardiac dysfunction. Two subjects were smokers. The mean of the BMI was 26.9 ± 3.9 kg/m² (range: 22.8‐34.3 kg/m²). The dorsal forearm of each subject was defined as the ROI.

### 2.3 Experimental protocol

**Figure 1:**
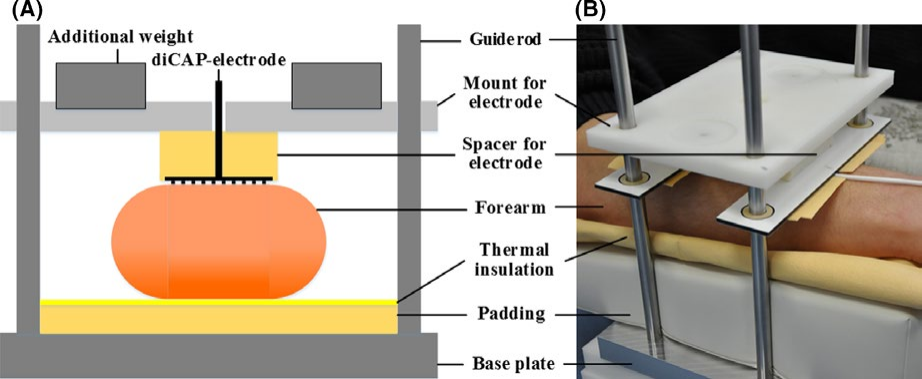
(A) Scheme of the customized mount for both reproducible plasma application and microcirculation measurements with an optical probe. (B) Photograph of the experimental setup with the optical probe (not visible) installed in the mount

It has been observed in preliminary tests and is also known from the literature that local thermal changes, body movements, level of attention, and emotional stress can affect microcirculation parameters of the dorsal forearm.^32^, ^33^, ^34^, ^35^ To address these issues and control their impact on microcirculation parameters throughout the studies, we developed a rigid experimental protocol (Table 1) and constructed a customized mount for diCAP electrode placement and probe positioning in the ROI as depicted in Figure 1. The probe was integrated in the mount in such a way that an even contact face could be realized and the weight pressure of the mount was evenly distributed across its complete area. The probe measures in an area of approx. 1 cm² centered in the mount (position corresponded to center position of the diCAP electrode). The mount allowed to press the optical probe for microcirculation assessment as well as the diCAP electrode reproducibly onto the ROI at a defined weight pressure (optical probe: 7 mm Hg (10 g/cm²), diCAP electrode: 96 mm Hg (130 g/cm²)) during every single experimental session. The applied pressure of the diCAP electrode was 96 mm Hg, which corresponds to the typical contact pressure applied during a standard diCAP treatment with the PlasmaDerm^®^FLEX9060‐device. The exact same measuring position was reproducible in every subject. Heat insulation was applied to the mount at every point of contact with the skin to minimize thermal irritations of the skin. To minimize experimental uncertainties due to body movements, the subjects were placed in supine position on a padded mattress and were advised to refrain from moving any part of the body during each experimental session of approx. 100 minutes. In preliminary studies, we encountered the problem that most of the subjects fell asleep during the experiment which changed microcirculation parameters substantially. Several possibilities for prevention were investigated, for example, concentrate on breathing, listening to audio books, music, and to radio plays. As the most effective option to prevent falling asleep combined with achieving a level of attention as continuous as possible, we finally decided to show movies to the subjects. The plots of the movies were well known to most of the subjects thus avoiding strong emotional reactions. As a consequence of all these measures, the procedures for diCAP treatment and microcirculation measurement were very reproducible.

**Table 1:** Consecutive actions and corresponding durations of the experimental protocol

#	Action	Duration	Data record
1	Measurement of skin temperature, pH, and moisture	5 min	
2	Stabilization of perfusion	10 min	
3	Measurement of microcirculation parameters in the ROI	10 min	Baseline
4	Application of the diCAP electrode without plasma treatment	90, 180, or 270 s	
5	Measurement of microcirculation parameters in the ROI	10 min	Pressure
6	Plasma treatment	90, 180, or 270 s	
7	Measurement of temperature in the ROI	30 s	
8	Measurement of microcirculation parameters in the ROI	60 min	Pressure + Plasma
9	Measurement of skin temperature, pH, and moisture	5 min	

The experimental protocol followed a strict time lapse with the actions given in Table 1. Each of the ten subjects passed a total of three times the entire experimental protocol—each time, however, with a different treatment period of 90 seconds, 180 seconds, or 270 seconds, respectively. At first, skin temperature, pH, and moisture were measured in the ROI (#1). After resting in supine position with the forearm positioned in a controlled position within the customized mount for 10 minutes (#2), the baseline data of the microcirculation parameters in the ROI were recorded for a period of 10 minutes (#3). Directly following these measurements, the diCAP electrode was placed on the ROI for 90 seconds, 180 seconds, or 270 seconds, respectively, but no plasma treatment was conducted (#4). As soon as the diCAP electrode was removed from the ROI, the microcirculation parameters were recorded once more for a period of 10 minutes (#5). Then, the diCAP treatment was performed for 90 seconds, 180 seconds, or 270 seconds, respectively (#6), followed by measuring the skin temperature (#7) followed by microcirculation assessment over a period of 60 minutes (#8). Finally, skin temperature, pH, and moisture were measured in the ROI (#9).

### 2.4 Measurement of microcirculation parameters and data processing

Microcirculation parameters were recorded by the noninvasive optical system Oxygen‐to‐see (O2C) (LEA Medizintechnik GmbH, Giessen, Germany). Conformity for the class IIa medical device with EU Guideline 93/42/EEC for medical devices is declared by the manufacturer. In brief, the O2C combines laser Doppler fluxmetry (820 nm, 30 mW) for the determination of blood flow and blood flow velocity in arbitrary units [A.U.] and spectrophotometry (450‐1000 nm, 20 W) for detection of SO2 in percentage [%] and local relative hemoglobin (rHb) in arbitrary units [A.U.]. In laser Doppler fluxmetry, the Doppler shift of erythrocytes in the tissue volume is detected and the product of moving erythrocytes *N*_*i*_ and the corresponding velocity *v*_*i*_ of each erythrocyte are used for the calculation of the relative blood flow via Equation (1):

(1) $$\text{Blood flow}=\sum v_{i}\cdot N_{i}$$

In spectrophotometry, white light is used for the determination of SO2 and rHb. The change in spectral distribution (color) of the reflected light is due to a wavelength‐dependent absorption of the applied white light and can be used for the calculation of SO2. In contrast, rHb correlates with the amount of light absorbed by the tissue (intensity). This measurement of rHb represents a hemoglobin concentration *n*_*e*_ per tissue volume, which is independent of the erythrocyte velocity, vessel density, vessel lumen, and hemoglobin quantity in the blood. The interested reader is referred to Forst et al^36^ for more details on the measurement technique.

For interpreting data from laser Doppler fluxmetry in the context of data from spectrophotometry, it is important to note that the procedure for determining *N*_*i*_ only takes into account erythrocytes with velocities *v*_*i*_ > 0, whereas the procedure for determining *n*_*e*_ considers all erythrocytes (*v*_*i*_ ≥ 0) and thus gives higher values.

For the measurements, a probe mounted on the tissue surface introduces light into the tissue and detects the backscattered signal. As stated by the manufacturer, the measuring depth of the applied probe LFX‐29 (LEA Medizintechnik GmbH) in this study is 1‐2 mm. The O2C system has been successfully applied before to measure diCAP‐induced changes in skin microcirculation.^28^, ^29^, ^30^

According to Table 1, the output of this study is three raw data records of time‐resolved (Δt = 25.6 ms) microcirculation parameters per subject and treatment time, which are indicated as Baseline, Pressure, and Pressure + Plasma. For time‐resolved microcirculation parameters, the raw data records with *i* data points were reduced to records with *j* data points at a temporal resolution of 30 seconds (*i *=* *1172) according to Equation (2):

(2) $${\overset{¯}{x}}_{j,\mathrm{time}-\;\mathrm{resolved}}=\frac{1}{1172}\sum_{i=1172\;j+1}^{1172\;(j+1)}x_{i}$$

For time‐averaged microcirculation parameters, raw data of the Baseline records and the Pressure records were averaged over the entire acquisition period of 10 minutes (*i *=* *23428) per subject and treatment duration according to Equation 3a. The Pressure + Plasma records were averaged over a period of 60 minutes (*i *=* *140625) per subject and treatment duration according to Equation 3b:

(3a) $${\overset{¯}{x}}_{\mathrm{time}-\;\mathrm{averaged},10\min}=\frac{1}{23428}\sum_{i=1}^{23428}x_{i}$$

(3b) $${\overset{¯}{x}}_{\mathrm{time}-\;\mathrm{averaged},60\min}=\frac{1}{140625}\sum_{i=1}^{140625}x_{i}$$

#### 2.4.1 diCAP application

For diCAP treatment, the class IIa medical device PlasmaDerm^®^FLEX9060 (CINOGY GmbH, Duderstadt, Germany) was used. Conformity with EU Guideline 93/42/EEC for medical devices is declared by the manufacturer. According to the manufacturer, the plasma area amounts to 27 cm².

The device features a single high‐voltage electrode that generates sufficiently high electric fields for air breakdown only as soon as it is pressed gently on a tissue surface (or other conductive material). Electrically, the tissue acts as the counter electrode and is part of the (secondary) electrical circuit of the device. This concept to create a CAP is also referred to as DBD and belongs to the group of direct plasma sources.^37^ For details on typical plasma process parameters, the interested reader is referred to literature.^38^ In this work, the power density was determined to be 4 mW/cm² by an approved measurement technique when the single high‐voltage electrode is operated against a metal counter electrode.^39^

### 2.5 Measurement of skin temperature, pH, and moisture

Skin pH was tested with the potentiometrically operating electrode Inlab Surface (Mettler Toledo GmbH, Giessen, Germany) measuring at an accuracy of ± 0.01. Skin moisture in percentage was measured with a MoistureMeterD compact (Delfin Technologies UK Ltd., Dorking, UK) with a measurement accuracy of ± 5%. The measuring principle is based on noninvasive detection of the tissue dielectric constant. Both instruments were in contact with the skin and especially the measurement of skin moisture needed contact pressure. Therefore, these measurements could have an influence on microcirculation parameters. Consequently, we measured these two parameters only before and after the experiment and thus chose a contact‐free infrared thermometer FT90 (Beurer medical GmbH, Ulm, Germany) at an accuracy of ± 2°C. In accordance with literature, there were no decisive temperature‐driven changes in blood flow under 35°C expected.^40^, ^41^ For this reason, we expected the accuracy was sufficient. The contact‐free measurement of skin temperature enabled this measurement during the experiment without an influence on microcirculation parameters. Data are provided as mean ± SD of all subjects (N = 10) per treatment duration and time of acquisition.

### 2.6 Statistical analysis

A statistical power analysis was carried out using the statistical software G*Power 3.1. Based on our previous experience, we estimated an effect size of 1.3; the probability of a type 1 error was set to 5% (alpha = 0.05) a power to 95% (beta = 0.95) this resulted in a necessary sample size of 10 with an actual power of beta = 0.954. The statistical analysis of experimental data was performed using the data management solution Origin^®^ 2015G (OriginLab Corp., Northampton, MA, USA) and SPSS (IBM, Armonk, NY, USA). Data are provided as mean ± SD of all subjects (N = 10) per treatment duration. For pH values, statistical significance was evaluated by a paired two‐sample *t* test, whereas microcirculation parameters were analyzed applying univariate ANOVA testing with repeated measures followed by Bonferroni post hoc analysis. A *P*‐value < .05 was regarded as statistically significant.

## 3 RESULTS

### 3.1 Adverse events

Throughout the experiments, three subjects reported a tingling sensation during plasma treatment which was interpreted as AE. However, these sensations did neither lead to premature termination of the plasma treatment nor the trial. No SAE were reported.

### 3.2 Skin temperature, moisture, and pH

**Figure 2:**
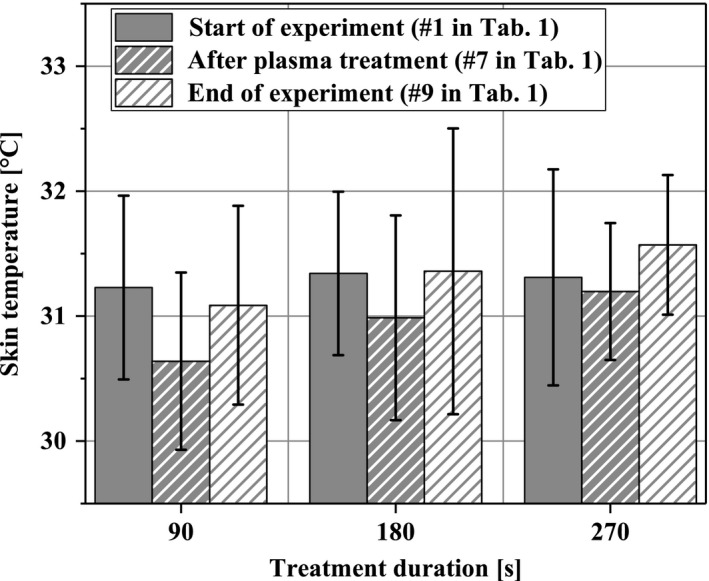
Skin temperature of the dorsal forearms at the beginning of the experimental protocol, immediately after plasma treatment and after completion of the experimental protocol

The surface temperatures in the ROI for treatment periods of 90 seconds, 180 seconds, and 270 seconds, respectively, are depicted in Figure 2. No significant variations in skin temperature could be observed due to plasma treatment within the range of experimental uncertainty (< 2°C).

**Figure 3:**
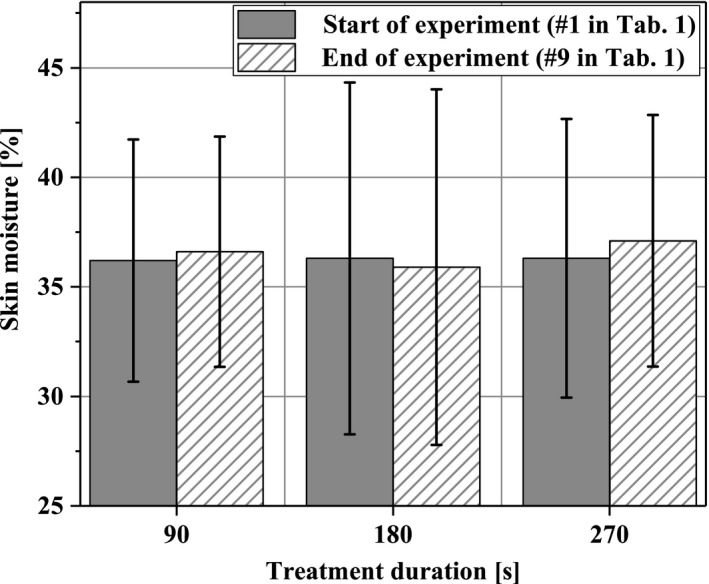
Skin moisture of the dorsal forearms before and after completion of the experimental protocol

Skin moisture in the ROI was relatively constant throughout the experiments and ranged from 35.0 to 37.5 before and after the experiments without significant changes (Figure 3).

**Figure 4:**
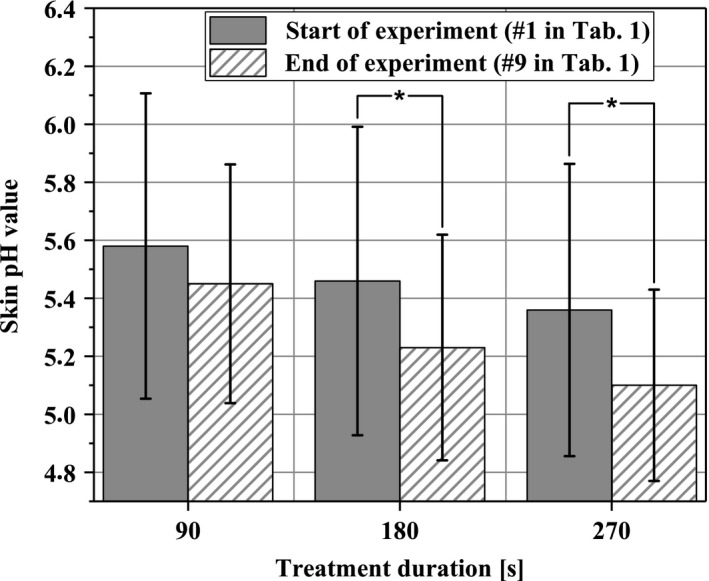
Skin pH value of the dorsal forearm before and after completion of the experimental protocol (*P < .05)

Figure 4 illustrates the skin pH values in the ROI. For all plasma‐treatment periods, the pH decreased by up to 0.3. For 180 seconds and 270 seconds plasma treatment, these changes were statistically significant.

### 3.3 Microcirculation

**Figure 5:**
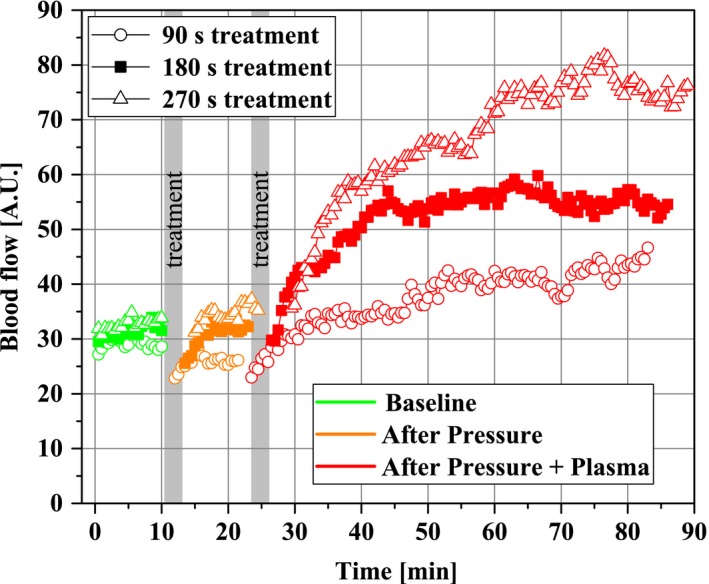
Time‐resolved characteristics of the cutaneous blood flow (N = 10) after stimulation with Pressure and with Pressure + Plasma for different treatment durations (indicated by gray bars). For clarity, SD data are only provided in Figure 9

In Figure 5, the time‐resolved blood flow characteristics are given as mean values of the subjects and visually separated by colors into the three microcirculation acquisition phases—Baseline (green), Pressure (orange), Pressure + Plasma (red)—of the experimental protocol. It becomes apparent that the blood flow responses to pressure stimulation are relatively mild compared to the responses to the Pressure + Plasma stimulation. There was a short decrease in blood flow immediately after induction of pressure. However, this change was reversed in the first 10 minutes after application through adaptation, resulting in a constant plateau that was not significantly different from the baseline. Blood flow changes induced by diCAP were much more pronounced and highly individual within the ten subjects. Some subjects show a constant increase over time, whereas the reaction of others followed an asymptotic behavior. Lastly, in some individuals, the blood flow increase is very strong but then tends to decrease within the acquisition period.

However, the mean values for all treatment durations including plasma application indicate an increase in blood flow—the longer the plasma‐treatment time, the more pronounced was the blood flow increase. It is worth noting that the predominant increase in blood flow did not occur immediately during the treatment, but minutes after the plasma was applied.

#### 3.3.1 Local relative hemoglobin (rHb)

**Figure 6:**
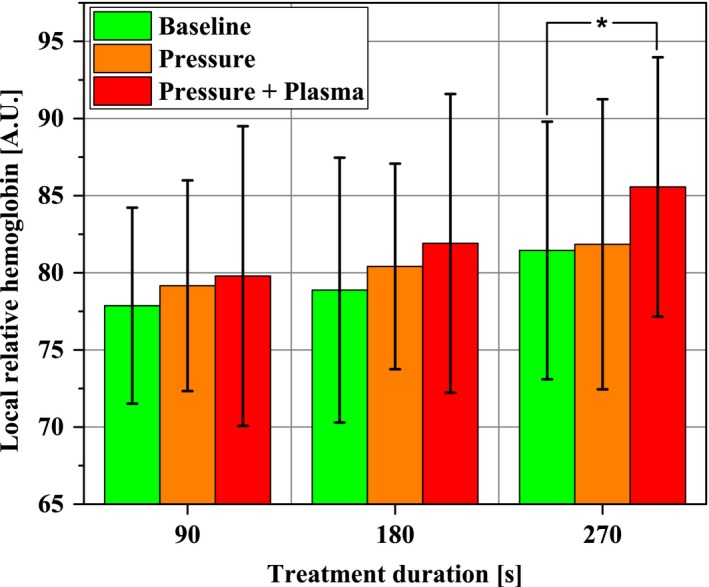
Local relative hemoglobin in the ROI (1‐2 mm depth) at the beginning of the experimental protocol (Baseline; time average of 10 min), after pressure induction by the diCAP electrode (Pressure; time average of 10 min), and after operating the diCAP electrode at constant power density (Pressure + Plasma; time average of 1 h) (*P < .05)

Within the time‐averaged means over 10 minutes and 1 hour, respectively, in Figure 6, only the Pressure + Plasma treatment for 270 seconds increased the rHb statistically significant by 5.1% from 81.5 ± 8.4 to 85.6 ± 8.4 for a period of 1 hour after plasma treatment whereas Pressure stimulus alone did not compared to no intervention. Significance levels between Pressure + Plasma and Pressure alone were checked to gain deeper insights into mechanisms of rHb increase. Yet, the Pressure + Plasma data were not significantly different from the mean values on sole Pressure stimulus.

#### 3.3.2 Blood flow velocity

**Figure 7:**
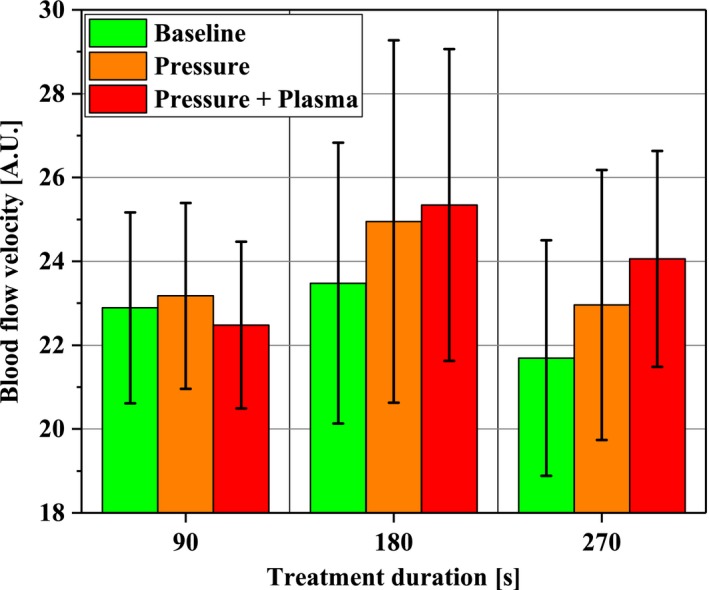
Blood flow velocity in the ROI (1‐2 mm depth) at the beginning of the experimental protocol (Baseline; time average of 10 min), after pressure induction by the diCAP electrode (Pressure; time average of 10 min), and after operating the diCAP electrode at constant power density (Pressure + Plasma; time average of 1 h)

The time‐averaged mean of blood flow velocity in Figure 7 showed no change after 90 seconds and 180 seconds, respectively, of Pressure or Pressure + Plasma. After 270 seconds treatment duration, an increase by trend was demonstrated only for Pressure + Plasma compared to no intervention (*P *=* *.052).

#### 3.3.3 Postcapillary oxygen saturation

**Figure 8:**
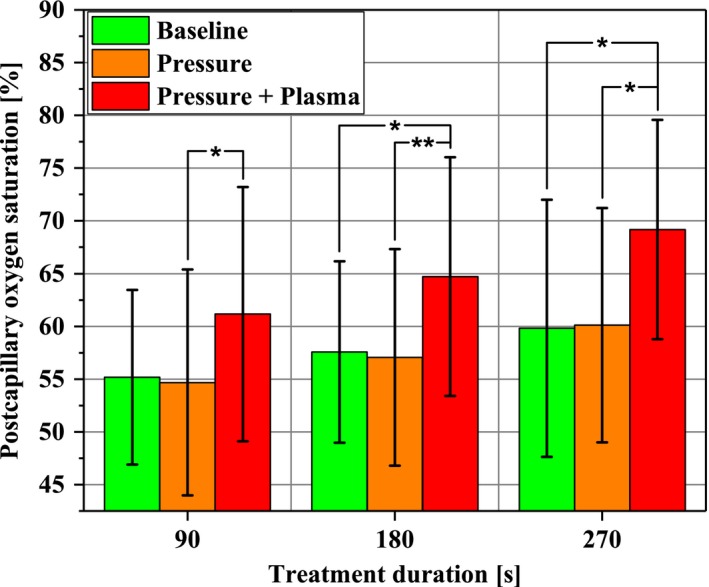
Tissue oxygen saturation in the ROI (1‐2 mm depth) at the beginning of the experimental protocol (Baseline; time average of 10 min), after pressure induction by the diCAP electrode (Pressure; time average of 10 min), and after operating the diCAP electrode at constant power density (Pressure + Plasma; time average of 1 h) (*P < .05, **P < .01)

The time‐averaged means for 10 minutes and 1 hour, respectively, for postcapillary oxygen saturation are given in Figure 8. Apparently, the sole Pressure stimulus for up to 270 seconds did not provoke alterations in the skin microcirculation compared to Baseline. In contrast, Pressure + Plasma intervention for 180 s significantly increased postcapillary oxygen saturation by 7.1% from 57.6 ± 8.6% to 64.7 ± 11.3%. With 9.4% from 59.8 ± 12.2% to 69.2 ± 10.4% after 270 seconds, this effect was even more pronounced. Once more, in order to elucidate the respective contributions of pressure or plasma stimulus to these increases, we analyzed significances between Pressure + Plasma and Pressure alone. Thereby, significant differences were found for all, 90 seconds (+6.5%), 180 seconds (+7.6%), and 270 seconds (+9.1%), respectively.

#### 3.3.4 Blood flow

**Figure 9:**
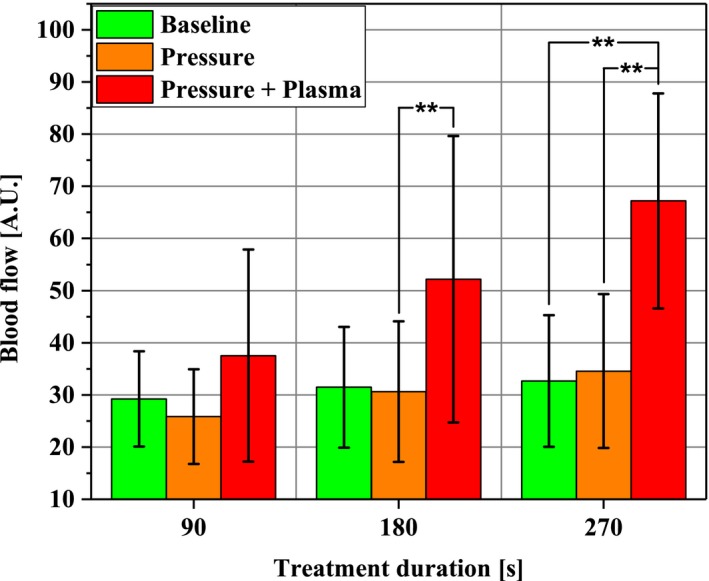
Blood flow in the ROI (1‐2 mm depth) at the beginning of the experimental protocol (Baseline; time average of 10 min), after pressure induction by the diCAP electrode (Pressure; time average of 10 min), and after operating the diCAP electrode at constant power density (Pressure + Plasma; time average of 1 h) (**P < .01)

Figure 9 depicts the response of cutaneous blood flow to stimuli for up to 270 seconds. Again, pressure‐induced effects did not lead to significant changes compared to Baseline. Yet, a significant increase by 105.5% from 32.7 ± 12.6 to 67.2 ± 20.6 over a 1‐h period occurred after diCAP treatment for 270 seconds. To analyze the particular contributions of pressure and plasma stimulus, comparison of Pressure + Plasma to sole Pressure data revealed an increase for 180 seconds treatment (+70.4%) as well as for 270 seconds treatment (+94.5%).

## 4 DISCUSSION

On a general note, it is worth mentioning that in accordance with the relevant literature no (S)AE was reported due to diCAP application on human tissue during our study. Positive effects on cutaneous microcirculation have already been observed.^28^, ^29^, ^30^ Yet, until now, it has been unclear, if the device‐induced pressure to the tissue should be considered a confounding factor in microcirculation enhancement by diCAP application. Therefore, in this study, the role of mechanical pressure induced by the diCAP device was addressed. Furthermore, it was so far unclear, how the tissue would react to longer treatment times with diCAP. Consequently, in this study, we applied diCAP for 90 seconds, 180 seconds, and 270 seconds, in a controlled mechanical environment.

It is well known, that pressure on the skin can influence skin perfusion. Whereas preclinical DBD sources can be operated without physical contact to the skin, most plasma medical products inherently exert a mechanical pressure on the treated tissue. Indeed, in the time‐resolved blood flow characteristics (Figure 5), an initial yet reversible occlusion after the pressure stimulus (96 mm Hg) becomes apparent. Autoregulative mechanisms compensate for this effect within less than 10 minutes. Within our experimental protocol, the occlusion effect is inevitably taken into account when calculating time‐averaged mean values after Pressure stimulus and leads to slightly decreased mean values for postcapillary oxygen saturation (Figure 8) and blood flow (Figure 9) compared to Baseline level. This effect could explain our counter‐intuitive observations that for 90 seconds in Figure 8 and for 180 seconds in Figure 9, we found significant differences comparing Pressure + Plasma data to sole Pressure data, whereas Pressure + Plasma data did not yield statistically significant differences when compared to Baseline results. In summary, the level of microcirculatory upregulation released by diCAP (Pressure + Plasma) was overall stronger compared to autoregulative recovery following pressure‐induced occlusion and present for a long time scale of at least 1 hour. Consequently, our first finding is that plasma‐induced effects during application of the diCAP source are dominant over mechanical pressure impact by the electrode for enhancing microcirculation.

According to literature, skin temperatures of the human forearm above 35°C show a decisive effect on blood flow.^40^, ^41^ Plasma heat transfer‐induced skin temperatures were well below this threshold, even taking the relatively low device accuracy for temperature measurements into account. Thus, our results on physiological skin parameters indicate that diCAP treatment for up to 270 seconds did not relevantly affect skin temperature. The same is true for skin moisture. Thus, we can rule out an impact of these variables on our results, which is important given that it has long been known that local temperature influences the peripheral blood flow.^42^ As our second finding, we state that in our experiments, temperature‐driven impact on microcirculation does not play a role.

The third finding is given by the observation that the skin pH significantly decreases with prolonged treatment durations. This general trend is well known for CAP generated in air and it is attributed to reactions of plasma‐generated gaseous species (reactive oxygen and nitrogen species—RONS) with omnipresent H_2_O, which lead to the formation of nitrous acid, nitric acid, and hydrogen peroxide.^43^, ^44^ In this context, it was hypothesized that NO penetration into the skin may be a possible mechanism for plasma‐induced changes of microcirculation.^27^, ^45^, ^46^

In general, for diCAP treatment durations of 90 seconds, 180 seconds, and 270 seconds, respectively, we observed a more pronounced impact on microcirculatory parameters with increasing treatment time indicated by an absolute increase in significance levels.

In general, quantitative results on microcirculation data induced by diCAP are to be interpreted in the context of the individual study design. In agreement with literature data, we confirm that diCAP enhances microcirculation for a much longer period than the application time. Heuer et al presented their results with a temporal resolution of 1‐20 minutes, whereas Kisch et al measured at 1 min resolution.^28^, ^29^, ^30^ Even though in our study, data were recorded at a temporal resolution of 25.6 ms, our results are given as mean values over a period of up to 60 minutes. The rationale of this approach is a rather practical focus on clinical impact and applicability in patient care.

Our results on local relative hemoglobin show an increase about 5.1% after 270 seconds of treatment for a period of 60 minutes. Heuer et al found an enhancement of 30% in rHb over a period of 60 minutes following a 90 seconds treatment, whereas Kisch et al observed no increase after a single application for 90 seconds. After 3 × 90 seconds treatment, they found the rHb significantly increased for 40 minutes by max. 12%.^28^, ^29^, ^30^

Concerning blood flow velocity, our study revealed no significant alterations due to diCAP treatment. Heuer et al observed a doubling (90 seconds treatment) after 5 minutes, which steadily decreases to a factor of 1.6 after 45 minutes.^28^, ^29^, ^30^

The postcapillary oxygen saturation was upregulated by 7.1% (180 seconds diCAP) and 9.4% (270 seconds diCAP) for 60 minutes. Heuer et al found an upregulation by 37% for 60 minutes after 90 seconds of treatment. Kisch et al found up to 24% increase for 8 minutes after single and up to 47% for 40 minutes after repetitive application.^28^, ^29^, ^30^ Statistical analysis clearly indicates that upregulation of oxygen saturation is driven by the physiological potential of the plasma and not affected by the implied electrode pressure on the skin.

Blood flow doubled following 270 seconds diCAP in our experiments for 1 hour. Once more, this increase is not caused by mechanical pressure impact. Heuer et al measured a roughly fourfold higher blood flow after 5 min, which decreased to threefold after 45 minues. Kisch et al found an increase up to 73% for 11 minutes after single treatment and an increase about a factor of up to 2.5 for a period of 12 minutes after repetitive treatment for 3 × 90 seconds.^28^, ^29^, ^30^

As the diagnostic device in our study is identical to the device applied in the literature, differences in quantitative results either derive from slight modifications (eg, mechanical pressure exerted by the diCAP device in comparison with the studies of Kisch et al, optical probe attachment on the skin) in the individual experimental protocols or from nonidentical diCAP process parameters. Indeed, the diCAP device applied in Heuer et al not only features a different material (ceramic instead of plastic) but also a significantly smaller geometry with at a diameter of no more than 10 mm. Furthermore, as can be judged from the provided electrical parameters, this electrode was operated by a different electronic device than in the studies of Kisch et al and our study. Unfortunately, in Heuer et al*,* the plasma power density, which can be considered one of the most important process parameters, is not given. Considering the relatively small plasma area in Heuer et al compared to the studies of Kisch et al and our experiments, the pulse energy as well as plasma power density can be assumed the highest in Heuer et al Therefore, the relatively high‐energy input into the skin as well as the plasma (typically leading to high RONS concentrations in the plasma volume) obviously leads to a very pronounced impact on dermal microcirculation. Kisch et al operated their diCAP device with an area of 22.5 cm² at 12 mW/cm² (when operated against a metal counter electrode), whereas in our study, the device with an electrode area of 27 cm² was operated at 4 mW/cm².^39^ Concluding the above findings, the applied plasma power density seems to correlate with quantitative differences in microcirculation enhancement and thus might be a key process parameter.

When interpreting our results of blood flow data and blood flow velocity data (see Equation (1)), we conclude that the number of moving erythrocytes *N*_*i*_ is disproportionally increased compared to their velocity *v*_*i*_ thus indicating the effect of a plasma‐induced vasodilatation. To test this hypothesis, further investigations on the physiological mechanisms of diCAP on microcirculation should be conducted.

Due to different morbidities associated with reduced perfusion (ie, Diabetes, arteriosclerosis, polyneuropathy, overweight), a growing number of patients suffer from impaired wound healing.^47^ In this context, we spare an elaborated discussion on the pharmaceutical therapy, as it is usually applied preventively or in early stage of wounds.^48^, ^49^ Surgical techniques predominantly address macrovascularization to restore blood flow. Yet, blood flow on a macrovascular level alone will not reverse microcirculatory derangements, which are critical in wound healing processes.^48^, ^50^ In clinical practice, perfusion data are commonly given as tcPO2 derived from arterial oxygen concentration.^51^ In contrast, the noninvasive and sensitive O2C system as diagnostic device for microcirculation gives information about venous postcapillary oxygen saturation. Consequently, tcPO2 and SO2 provide different information and can thus not be compared quantitatively. In fact, SO2 data can provide substantial additional information and thus assist physicians in assessing clinical cases.

An important issue arises from the question, to what extent the effect of microcirculation enhancement by diCAP in healthy subjects is transferable to patients with impaired tissue. As a matter of fact, this question needs to be addressed in studies facilitating a cohort of patients with defined morbidities. As of now, we can only hypothesize on the basis of available literature. Beckert et al^52^ were the first group evaluating the O2C device. They grouped subjects with healthy skin and those with diabetic ulcers. In the latter, they defined a subgroup of healers and nonhealers. Their results showed that within the nonhealers group blood flow, rHb, and SO_2_ were significantly lower compared to the healer subgroup, whereas tcPO2 values (27 ± 6.0 mm Hg vs 19 ± 7.3 mm Hg) did not reveal significant differences between healers and nonhealers. The authors concluded that tcPO2 is not an optimal parameter for evaluation of oxygen supply of the wound. Furthermore, they found that venous postcapillary oxygen saturation was significantly lower in the nonhealer group compared with healthy control subjects, whereas there was no difference between control subjects (healthy skin) and healers. At the wound site, nonhealers had significantly lower mean values in postcapillary oxygen saturation (50% vs 73%), local relative hemoglobin (54 A.U. vs 77 A.U.), and especially blood flow (19 A.U. vs 150 A.U.) compared to subjects with healing wounds. Surprisingly, comparing the quantitative results of microcirculation parameters derived from intact skin of healthy volunteers with data from the nonhealer subgroup in the wound group, it was found that they match quite well. This indicates no enhanced microcirculation at the wound site of nonhealers compared to healers.^52^ From these observations, we hypothesize that the diCAP‐induced enhancement of microcirculation observed on intact skin of healthy volunteers in this study might be transferable to future diCAP application at the wound site of nonhealers. This appears especially desirable in view of the strong blood flow increase induced by diCAP as blood flow was found to be extremely low in the wound site of nonhealers.^52^ Yet, upcoming studies must be performed to test this hypothesis.

With regard to clinical relevance, blood flow can be considered the driving parameter for microvascular oxygen supply, as postcapillary oxygen saturation and local relative hemoglobin are primarily a result of alterations in blood flow. Applying diCAP in a topical, noninvasive treatment doubles the blood flow. Saucy et al^53^ increased skin blood flow of fore‐ and hindfood after bypass and endarterectomy by 50% and 40%, respectively, measured in perfusion units as derived by a laser Doppler imaging system. Taking into account that diCAP is a noninvasive intervention and so far no serious side effects have been reported, it can be considered an innovative and competitive therapy option—particularly for poor candidates for surgical or endovascular procedures, whose comorbidities and poor outflow vessels limit revascularization as a viable option.^48^

We did not observe a declining tendency of elevated blood flow after 1 hour of diCAP treatment referring to the time‐resolved blood flow data. Yet, it is a nonpermanent effect in healthy subjects. In view of promising results on the induction of neo‐angiogenetic and epithelial growths effects by CAP treatment, we hypothesize that for the repetitive treatment of wounds the increase in blood flow might become a sustainable positive effect on vascular autoregulation in impaired tissue.^24^, ^54^, ^55^

## PERSPECTIVE

The results of the present investigation provide a new therapeutic approach to sustainably enhance microcirculation in cutaneous tissue at no side effects. Consequently, diCAP emerges as a new clinically promising option to restore impaired tissue and to assist wound healing. Furthermore, diCAP might assist physicians in the prevention of impaired wound healing in potential risk patients.
